# Geometrical Isotope Effects on Chemical Bonding in Hydrogen Bonded Systems: Combining Nuclear‐Electronic Orbital DFT and Energy Decomposition Analysis

**DOI:** 10.1002/jcc.70226

**Published:** 2025-09-11

**Authors:** Raza Ullah Khan, Ralf Tonner‐Zech

**Affiliations:** ^1^ Wilhelm‐Ostwald‐Institut für Physikalische und Theoretische Chemie Leipzig University Leipzig Germany

**Keywords:** charge‐inverted hydrogen bond, energy decomposition analysis, isotope effects, NEO‐DFT, nuclear quantum effects

## Abstract

We investigated primary and secondary geometric isotope effects (H, D, T) on charge‐inverted hydrogen bonds (CIHB) and dihydrogen bonds (DHB) using nuclear‐electronic orbital density functional theory (NEO‐DFT). The dianionic but electrophilic boron cluster [B_12_H_12_]^2−^ served as a bonding partner, exhibiting a negatively polarized hydrogen atom in the B—H bond. CIHB systems included interactions with Lewis acids (AlH_3_, BH_3_, GaH_3_) and carbenes (CF_2_, CCl_2_, CBr_2_), while DHBs were analyzed with NH_3_, HF, HCl, and HBr. Isotope substitution systematically decreased intermolecular and intramolecular bond lengths (H > D > T). Energy decomposition analysis (EDA) combined with Hirshfeld partial charge analysis confirmed the bonding interpretation but revealed significant variations in bonding contributions across different complexes. While some systems exhibited increased electrostatic attraction, others showed enhanced orbital interactions or shifts in Pauli repulsion, which could stabilize or destabilize the interaction. Natural orbital for chemical valence (NOCV) analysis highlighted charge depletion from the partially negative hydrogen towards the vacant orbital of the bonding partner in CIHB systems, further supporting the bonding model. This study demonstrates how isotope substitution influences electronic structure and lays the groundwork for extending such analyses to more strongly bound systems, where isotope effects may be more pronounced.

## Graphical abstract

We used nuclear electronic orbital density functional theory (NEO‐DFT) to investigate nuclear quantum effects on the atomic structure in charge‐inverted‐bonded and dihydrogen‐bonded systems. Beyond hydrogen, its heavier isotopes deuterium and tritium were analyzed. Further, energy decomposition analysis (EDA) was used to check the impact of geometric isotope effects on chemical bonding and disentangle the different bonding contributions.

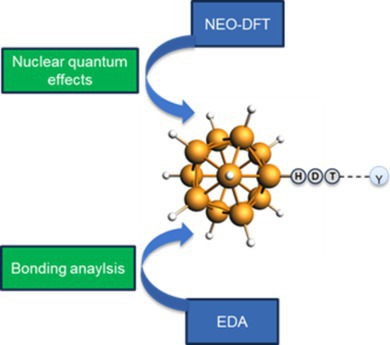

## 1 Introduction

Chemical bonding concepts are central to the discussion of properties and reactivity in chemistry [1, 2, 3, 4, 5, 6]. The understanding has continuously evolved, starting from classical concepts like Lewis structures to modern approaches using valence bond or molecular orbital theory. Since then, the chemical bond has always been a controversially discussed and inspiring topic [7, 8, 9, 10, 11, 12]. As bonding is an inherently quantum mechanical effect, analyses naturally focus on the electronic structure of molecules and materials. However, the quantum nature of the nucleus can play a significant role in effects like tunneling or zero‐point vibrational energy. This is specifically true for light nuclei, especially hydrogen. But the quantum nature of nuclei also indirectly influences the chemical bonding properties, which is the focus of this study. The major consequence when moving from hydrogen (H) to deuterium (D) and tritium (T) is the geometric isotope effect, which has been studied in molecular systems before [13, 14, 15]. It was found that the heavier isotope substitution causes shrinkage of intramolecular distances (primary geometric isotope effects) which can be explained by the anharmonicity of the potential [15]. However, isotope effects on other intermolecular distances in chemical systems (secondary geometric isotope effects) still remain not well explored.

The analysis of hydrogen isotope substitution is a specifically instructive case since the number of nucleons doubles and triples when moving from H to T, thus a large effect is found. Well studied is the isotope effect on the properties of water: [16, 17, 18] The vaporization energy of normal water (H_2_O) is lower compared to heavy water (D_2_O) [19, 20]. However, beyond the case of water, few studies exist on isotope effects on chemical bonding [13, 14, 15, 21, 22]. But also in other materials, significant effects are found. For example, deuterium substitution significantly changes the phase transition temperature in ferroelectric materials [23]. It is not only the physical properties, but isotope substitution also affects the bonding situation in chemical systems. For example, hydrogen bonding in light water results in approximately 4% shorter bonds compared to heavy water [16, 21, 24, 25].

The large variations in atomic mass when moving from hydrogen (1.0073 u) to deuterium (2.0141 u) to tritium (3.0160 u) also lead to large changes in vibrational energy and thus in zero‐point energy (ZPE). Molecular vibrations are inversely proportional to the mass of the nuclei (implicitly assuming the Born–Oppenheimer approximation, BOA). Thus, hydrogen isotope substitution leads to a significant change in the vibrational frequencies in dihydrogen isotopologues (*ν*(H_2_) = 4161.2 cm^−1^, ν(HD) = 3632.2 cm^−1^ and *ν*(D_2_) = 2993 cm^−1^) [26] and consequently a change in ZPE, which is given as *E*(ZPE) = ½ *hν*. This means that isotope substitution directly impacts the strength of the bonding. Quantum mechanical effects such as tunneling arise from the inherently quantum nature of the atoms [27]. In such cases, it can become necessary to treat light nuclei at the same quantum level as electrons. There are several methods which can treat nuclei quantum mechanically [28, 29, 30, 31, 32, 33]. One of such methods is the nuclear electronic orbital density functional theory (NEO‐DFT) method. In NEO‐DFT, isotope effects can be captured comprehensively and in a computationally efficient way without invoking semi‐empirical approximations like WKB (Wentzel/Kramers/Brioullin) theory [34].

**SCHEME 1:**
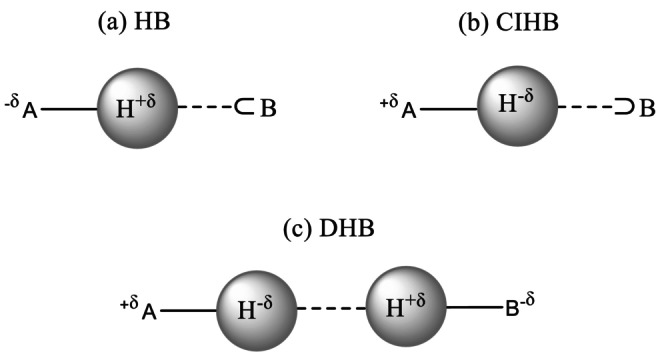
Different types of hydrogen bonds: (a) normal hydrogen bond (HB), (b) charge‐inverted hydrogen bond (CIHB) and (c) dihydrogen bond (DHB).

But which molecular systems are good starting points for investigating such isotope effects on chemical bonding? In the research field concerned with non‐covalent interactions, negatively charged hydrogen atoms interacting with adjacent units have raised attention in the recent past [35, 36, 37, 38, 39, 40, 41]. Scheme 1 shows different types of intermolecular bonds. The “standard” hydrogen bond (HB) results from the interaction between the partially positively charged hydrogen of the proton donor A^−δ^‐H^+δ^ and a lone pair donor ⊂B [38]. In most cases, both A and B are more electronegative than hydrogen (normal hydrogen bond). A similar interaction can also arise between negatively charged hydrogen atoms in A^+δ^‐H^−δ^ units and a Lewis acidic species ⊃B. Such kinds of intermolecular interactions are known as charge‐inverted hydrogen bonds (CIHB), where the charge shifts from hydrogen towards electron‐deficient species B [36]. It has been found theoretically that CIHB exist between Group 3 hydrides and Group 4 hydrides (R^X^_3_XH‐‐‐⊃YR^Y^_3_^−^ where X = Si or Ge and Y = B, Al or Ga and R^X^ = H, Cl, Me; R^Y^ = H, F, Cl, Me) [37, 38, 42]. Moreover, divalent carbene may exist in a low spin state when two high electron‐withdrawing groups (such as halogens) are attached to it, making one p orbital vacant, giving negatively charged hydrogen an opportunity to shift its electron density to the vacant p orbital on the singlet carbenes [43]. Similarly, partially negatively charged hydrogen atoms can interact with partially positively charged hydrogen atoms on another unit too, to form dihydrogen bonds (DHB) A^+δ^‐H^−δ^‐‐‐H^+δ^‐B^−δ^ [44]. Dihydrogen bonding has been studied previously and has been found to exist in systems such as (BH_3_NH_3_)_2_ [45] and H_3_SiH…HF [35].

Due to electronegativity, only a few chemical elements can form A—H bonds with negatively charged hydrogen atoms. Here, covalently bonded systems are even more rare, with strongly ionic systems (e.g., LiH) being the more general case. One specifically interesting set of compounds is boron clusters such as [B_12_H_12_]^2−^ which received considerable attention in the recent literature due to their unusual bonding behavior as electrophilic dianions [46, 47, 48, 49, 50]. They will be used here as species A in CIHB, as well as in DHB complexes. Previously, nuclear isotope effects on CIHB have been studied in H_3_XH‐‐‐YH_3_ (X = C, Si, Ge; Y = B, Al, Ga) by Udagawa and Tachikawa using the multicomponent MO method with MP2 [22]. We now use NEO‐DFT to investigate this question, extending the number of chemical systems analyzed.

**SCHEME 2:**
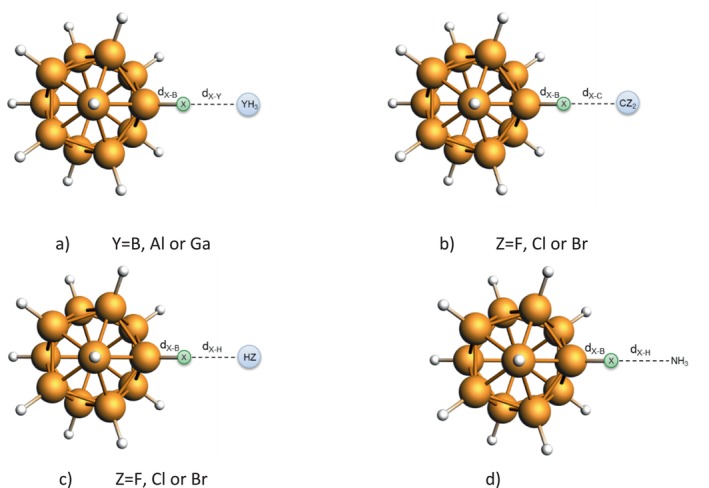
Representation of the CIHB (a, b) and DHB (c, d) complexes investigated in this study with important bonding parameters marked (X = H, D, T). Y=B, Al or Ga, (b) Z = F, Cl or Br, and (c) Z = F, Cl or Br.

To computationally investigate CIHB, we chose typical Lewis acids (YH_3_ with Y = B, Al, Ga) as well as strongly accepting singlet carbene species (CZ_2_ with Z = F, Cl, Br) as bonding counterparts (Scheme 1b). For the DHB, HF and NH_3_ were chosen as species B—H (Scheme 1c). We investigated CIHBs and DHBs shown in Scheme 2 with the NEO‐DFT method examining all hydrogen isotopes for the atom bonded to A (H, D, T). This enables us to quantify the isotope effect on chemical bonding in these hydrogen‐bonded complexes. We analyzed the resulting structures with the help of energy decomposition analysis (EDA) to quantify the changes in bonding resulting from isotope substitution. Although we only catch the geometric isotope effect with this approach, it is a first step toward rationalizing the influence of isotope substitution on chemical bonding in a larger set of molecules.

## 2 Methods

### 2.1 Theory of NEO‐DFT

In NEO‐DFT, protons are treated at the same level as electrons [34]. The total energy of a system in this approach can be written as a functional of protonic (*ρ* ^p^) and electronic densities (*ρ* ^e^):

(1) $$\begin{array}{c} E\left\lfloor \rho^{e}\rho^{p}\right\rfloor =\left(T_{s}\left[\rho^{e}\right]+T_{s}\left[\rho^{p}\right]\right)+\left(V_{\mathrm{ext}}^{e}\left[\rho^{e}\right]+V_{\mathrm{ext}}^{p}\left[\rho^{p}\right]\right)\\ \;+\left(J_{\mathrm{ee}}\left[\rho^{e}\right]+J_{\mathrm{pp}}\left[\rho^{p}\right]+J_{\mathrm{ep}}\left[\rho^{e},\rho^{p}\right]\right)\\ \;+\left(E_{\mathrm{xc}}^{e}\left[\rho^{e}\right]+E_{\mathrm{xc}}^{p}\left[\rho^{p}\right]+E^{e\mathrm{pc}}\left[\rho^{e},\rho^{p}\right]\right) \end{array}$$

Where the terms in Equation (1) are: the non‐interacting kinetic energy of the electrons $T_{s}\left[\rho^{e}\right]$ and protons $T_{s}\left[\rho^{p}\right]$; the interaction of the external potential with the electrons $V_{\mathrm{ext}}^{e}\left[\rho^{e}\right]$ and protons $V_{\mathrm{ext}}^{p}\left[\rho^{p}\right]$; the electron–electron $J_{\mathrm{ee}}\left[\rho^{e}\right]$, proton‐proton $J_{\mathrm{pp}}\left[\rho^{p}\right]$, and electron‐proton $J_{\mathrm{ep}}\left[\rho^{e},\rho^{p}\right]$ mean field Coulomb interactions; the exchange‐correlation energies of the electrons $E_{\mathrm{xc}}^{e}\left[\rho^{e}\right]$ and protons $E_{\mathrm{xc}}^{p}\left[\rho^{p}\right]$; and the electron‐proton correlation energy $E^{\mathrm{epc}}\left[\rho^{e},\rho^{p}\right]$. The formalism by Kohn and Sham can be used for Equation (1) to determine electronic densities, protonic densities, and the total energy of the system. Within the NEO‐DFT framework, the existing electronic exchange correlation functionals (the ones being used in non‐NEO DFT computations) can be used. Due to the spatial localization of the quantum proton, the proton‐proton exchange and correlation are minute and are usually neglected (also here). However, proper electron proton correlation is required for obtaining good protonic densities and total energies. So far, few electron proton correlation functionals have been proposed such as epc17 [51] and epc‐19 [52]. The epc17 functional is of local density approximation (LDA) type, and its mathematical form is given by:

(2) $$E_{\mathrm{epc}17}\left[\rho^{e},\rho^{p}\right]=-\int \frac{\rho^{e}\left(r\right)\rho^{p}\left(r\right)}{a-b{\left[\rho^{e}\left(r\right)\rho^{p}\left(r\right)\right]}^{\frac{1}{2}}+c\rho^{e}\left(r\right)\rho^{p}\left(r\right)}\;\mathrm{dr}$$

where *a*, *b*, and *c* are parameters. The values *a*, *b*, and *c* for the second available parametrization of this functional (epc17‐2) are 2.35, 2.4, and 6.6 respectively, which were determined by fitting to the ZPEs of FHF^−^ and HCN molecules.

The epc‐19 functional is of generalized gradient approximation (GGA) type, which not only depends on the electronic and protonic densities but also on their gradient and contains two more parameters d and k.

$$E_{\mathrm{epc}19}\left[\rho^{e},\rho^{p},\nabla \rho^{e},\nabla \rho^{p}\right]=-\int \frac{\rho^{e}\left(r\right)\rho^{p}\left(r\right)}{a-b{\left[\rho^{e}\left(r\right)\rho^{p}\left(r\right)\right]}^{\frac{1}{2}}+c\rho^{e}\left(r\right)\rho^{p}\left(r\right)}\mathrm{dr}$$

(3) $$\begin{array}{c} \{1-d\left(\frac{{\left[\rho^{e}\left(r\right)\rho^{p}\left(r\right)\right]}^{-\frac{1}{3}}}{{\left(1+m_{p}\right)}^{2}}\times \left[m_{p}^{2}\frac{\nabla^{2}\rho^{e}\left(r\right)}{\rho^{e}\left(r\right)}-2m_{p}\frac{\nabla \rho^{e}\left(r\right).\nabla \rho^{p}\left(r\right)}{\rho^{e}\left(r\right)\rho^{p}\left(r\right)}+\frac{\nabla^{2}\rho^{p}\left(r\right)}{\rho^{p}\left(r\right)}\right]\right)\\ \exp\left[\frac{-k}{{\left[\rho^{e}\left(r\right)\rho^{p}\left(r\right)\right]}^{\frac{1}{6}}}\right]\} \end{array}$$

### 2.2 Energy Decomposition Analysis (EDA)

Energy Decomposition Analysis (EDA) [53, 54] dissects the total bond energy (ΔE_bond_) between two interacting fragments, A and B, into distinct and physically intuitive components. This approach gives a detailed idea about the nature of the bonding situation. The overall bond energy (Δ*E*_bond_), as expressed in Equation (4), is given as the sum of the interaction energy (Δ*E*_int_) and the preparation energy (Δ*E*_prep_).

(4) $${\mathrm{\Delta E}}_{\text{bond}}={\mathrm{\Delta E}}_{\mathrm{int}}+{\mathrm{\Delta E}}_{\text{prep}}$$

The term *E*_prep_ is the energy needed to distort both fragments A and B from their relaxed structures to match the structures they adopt within the complex. Δ*E*_int_ characterizes the energy gain upon the combination of these prepared fragments A and B, leading to the formation of the complex A‐B. Furthermore, within the framework of EDA, Δ*E*_int_ can be decomposed into electrostatic energy (Δ*E*_elstat_), Pauli repulsion (Δ*E*_Pauli_), and orbital energy (Δ*E*_orb_) (Equation 5).

(5) $$\Delta E_{\mathrm{int}}=\Delta E_{\text{elstat}}+\Delta E_{\text{pauli}}+\Delta E_{\mathrm{orb}}$$

The electrostatic energy term typically is of attractive nature, representing the alteration in energy due to the interaction of the unperturbed charge distributions of the prepared fragments. In contrast, the Pauli repulsion term is the force of repulsion between the occupied fragments stemming from obeying the Pauli principle. Finally, the orbital energy term accounts for charge transfer and polarization effects. More details of the method can be found in the literature [55, 56].

In some cases, the numbers will not add up exactly as indicated by the equations above due to rounding. More details on the bonding nature are obtained from EDA‐NOCV analysis, which decomposes the deformation density between the intermediate and final states of the Δ*E*_orb_ step [57]. The density difference can be represented by Natural Orbitals for Chemical Valence (NOCV) which allow for further interpretation. The NOCVs result as a pairwise set with the same eigenvalues (with different signs) and only a few pairs are relevant (i.e., have high eigenvalues). Furthermore, the ETS scheme allows for the decomposition of the orbital interaction term in contributions from these NOCVs:

(6) $$\Delta E_{\mathrm{orb}}=\sum_{K=1}^{N/2}{\mathrm{\Delta E}}_{K}^{\mathrm{orb}}=\sum_{K=1}^{N/2}\upsilon_{K}\left[{-F}_{-K,-K}^{\mathrm{TS}}+F_{K,K}^{\mathrm{TS}}\right]$$

In the above Equation (6), ${-F}_{-K}^{\mathrm{TS}}$ and $F_{K}^{\mathrm{TS}}$ are the diagonal elements of the Fock matrix at the transition state between intermediate and final wavefunction with eigenvalues of $-\upsilon_{K}$ and $\upsilon_{K}$ respectively. For further details, readers are referred to the relevant articles for details and applications of EDA‐NOCV [56, 58, 59, 60].

### 2.3 Computational Details

All calculations were performed with Q‐Chem 6.0 [61] and ADF AMS (Amsterdam Density Functional, version 2021.104) [62] packages. All structures were optimized, and frequency calculations were performed in Q‐Chem. To reflect the nuclear quantum effects (NQEs) on the electronic structure, structure optimizations were performed with NEO‐DFT [34] at B3LYP/def2‐TZVP/EPC17‐2/4s4p4d level. This set of parameters and settings was found to be the most efficient in our previous study [63]. We used the GDM (geometry direct minimization) [64] algorithm and an electronic energy convergence criterion of 10^−11^ Ha. Standard settings of convergence criteria were used for geometry optimization. To capture isotope effects on chemical bonding, EDA‐NOCV calculations were performed on the optimized structures using the ADF package with B3LYP/TZ2P (without NEO) applying scalar relativistic effects and using a small frozen core. The same setup was used for Hirshfeld charge calculations.

## 3 Results

### 3.1 Geometric Isotope Effects

First, we discuss the CIHB systems (Scheme 2a,b). Table 1 shows some key bonding parameters. The results reveal a consistent trend of shrinking intermolecular (*d*_X‐Y/Z/H_) and intramolecular distances (*d*_X‐B_) with isotopic substitution—bonds involving X = H are longer than for *X* = D, which are in turn longer than for X = T. For example, for the [B_12_H_11_X]^2−^‐BH_3_ complex, the intramolecular bond length (*d*_X‐B_) is 1.290 Å (X = H) which successively decreases to 1.283 Å (X = D) and 1.280 Å (X = T). The intermolecular bond distance (*d*_X‐Y_) for this complex is 1.387 Å (X = H) which also shrinks to 1.382 Å (X = D) and 1.377 Å (X = T) after deuterium and tritium substitution, respectively. Notably, this trend holds true for all CIHB systems except [B_12_H_11_X]^2−^‐GaH_3_ where *d*_X‐B_ does not change from X = D to X = T. The reduction in intramolecular bond distance finds its rationale in the differences in zero‐point energy among the isotopes and the potential anharmonicity. NEO‐DFT intrinsically captures these primary geometric isotope effects within the optimization process, which is then reflected in the shortening of bonds [13, 65]. We can thus quantify primary (intramolecular) as well as secondary (intermolecular) isotope effects here. Heavier hydrogen isotopes are more electronegative than the lighter ones [66] therefore carry a higher magnitude of the negative charge, which makes the boron cluster interact more strongly with the other molecules compared to the boron cluster with lighter isotopes. Isotope substitution also affects the bond angle; however, no consistent trend was found, and the changes are small. Our geometric isotope effects of shrinkage in both intermolecular and intramolecular distances are consistent with the previous work of Udagawa and Tachikawa on CIHB systems [22].

**TABLE 1:** The intermolecular (*d*_X‐Y/Z/H_) and intramolecular distances (*d*_X‐B_) in Å, and bond angles in degrees for all the complexes shown in Scheme 2.

X=	B = YH_3_	*d*_X‐Y_	*d*_X‐B_	*θ*_B‐X‐Y_
H	BH_3_	1.387	1.290	137.0
D	BH_3_	1.382	1.283	136.8
T	BH_3_	1.377	1.280	137.6
H	AlH_3_	1.800	1.267	133.2
D	AlH_3_	1.792	1.261	134.4
T	AlH_3_	1.787	1.258	135.1
H	GaH_3_	1.813	1.274	165.0
D	GaH_3_	1.809	1.267	164.6
T	GaH_3_	1.804	1.267	165.0

	B = CZ_2_	*d*_X‐Z_	*d*_X‐B_	*θ*_B‐X‐Z_
H	CF_2_	1.529	1.308	163.3
D	CF_2_	1.525	1.301	163.8
T	CF_2_	1.522	1.298	164.0
H	CCl_2_	1.255	1.461	169.1
D	CCl_2_	1.249	1.456	170.7
T	CCl_2_	1.245	1.454	171.0
H	CBr_2_	1.240	1.501	177.5
D	CBr_2_	1.234	1.496	176.9
T	CBr_2_	1.230	1.493	176.5

	B = HZ	*d*_X‐H_	*d*_X‐B_	*θ*_B‐X‐H_
H	HF	1.432	1.235	174.5
D	HF	1.425	1.230	174.5
T	HF	1.421	1.226	174.6
H	HCl	1.362	1.245	174.6
D	HCl	1.361	1.239	174.5
T	HCl	1.361	1.236	174.6
H	HBr	1.218	1.262	175.4
D	HBr	1.210	1.256	175.4
T	HBr	1.205	1.252	175.4

	B = NH_3_	*d*_X‐H_	*d*_X‐B_	*θ*_B‐X‐H_
H	NH_3_	2.280	1.230	121.9
D	NH_3_	2.275	1.224	122.1
T	NH_3_	2.271	1.220	122.3

For the DHB systems (Scheme 2c,d) also a consistent shrinkage in the intermolecular (*d*_H‐H_ > *d*_D‐H_ > *d*_T‐H_) and intramolecular (*d*_H‐B_ > *d*_D‐B_ > *d*_T‐B_) distances upon heavier isotope substitution is found (Table 1). Such as in the case of [B_12_H_11_X]^2−^‐HF, the intermolecular distance d_X‐H_ is 1.432 Å (X = H) which reduces to 1.425 Å (X = D) and 1.421 Å (X = T) upon deuterium and tritium substitution. For one case (B = HCl), no bond shrinkage is found in moving from X = D to X = T. Similarly, for the intramolecular distance d_X‐B_ in [B_12_H_11_X]^2−^‐HF, the distance is 1.235 Å (X = H) which shrinks to 1.230 Å (X = D) and 1.226 Å (X = T). Like for the CIHB systems, no clear trend of isotope substitution on bond angle was found.

### 3.2 Geometric Isotope Effects on Chemical Bonding

To understand the geometric hydrogen isotope effects on chemical bonding of the given complexes, EDA calculations were performed. For the [B_12_H_11_X]^2−^‐YH_3_ complexes (X = H, D, T; Y = B, Al, Ga) the EDA results are shown in Table 2. We will first discuss the trends for the series of group 13 Lewis acids and then turn to analyzing the isotope effects. The bond strengths follow the trend Al > B > Ga. The partial charges shown in Table 3 also show that the YH_3_ fragments accept charge as expected for a Lewis acid. However, the trend is here B > Al > Ga. This shows that simply taking charge transfer as an indicator of Lewis acidity can be misleading.

**TABLE 2:** EDA‐NOCV results (B3LYP/TZ2P) for [B_12_H_11_X]^2−^‐YH_3_ complexes.

X=	Y = B			Y = Al			Y = Ga		
	H	D	T	H	D	T	H	D	T
		Δ*E* ^a^	Δ*E* ^a^		Δ*E* ^a^	Δ*E* ^a^		Δ*E* ^a^	Δ*E* ^a^
Δ*E*_int_	−152.5	0.3	0.1	−170.8	0.5	0.8	−131.6	0.7	0.8
Δ*E*_Pauli_	253.3	3.1	4.4	143.4	1.1	1.7	155.1	1.2	3.6
Δ*E*_elstat_ ^b^	−146.1 (36%)	−1.1	−0.9	−167.5 (53%)	0.1	0.1	−153.1 (53%)	−0.4	−1.6
Δ*E*_orb_ ^b^	−259.7 (64%)	−1.7	−3.4	−146.6 (47%)	−0.7	−1.1	−133.6 (47%)	−0.1	−1.1
Δ*E*_1_ ^c^	−229.8	−1.3	−3.0	−109.1	−0.6	−1.0	−109.6	0.1	−0.7
Δ*E*_2_ ^c^	−16.1	−0.1	−0.3	−15.6	−0.2	−0.3	−12.4	−0.1	−0.3
Δ*E*_rest_ ^c^	−13.8	−0.3	−0.1	−22.0	0.2	0.3	−11.6	−0.1	−0.1
Δ*E*_prep_	54.1	−0.5	−0.4	49.3	−0.8	−1.2	36.5	−0.8	−1.0
Δ*E*_bond_	−98.4	−0.2	0.3	−121.8	−0.3	−0.4	−95.1	−0.1	−0.2

		Δ*d* ^d^	Δ*d* ^d^		Δ*d* ^d^	Δ*d* ^d^		Δ*d* ^d^	Δ*d* ^d^
*d*_H‐Y_	1.387	−0.005	−0.010	1.800	−0.008	−0.013	1.813	−0.004	−0.009

^a^ Δ*E* = *E*(D/T) – *E*(H). All energy terms in kJ·mol^−1^.

^b^ The percentage values give the contribution to the total attractive interaction Δ*E*_elstat_ + Δ*E*_orb_.

^c^ Contributions to Δ*E*_orb_ from EDA‐NOCV analysis (see Figure 1).

^d^ Δ*d* = *d*_D/T‐X_ – *d*_H‐X_. Distances in Å.

**TABLE 3:** Partial charges (q(Hirshfeld), B3LYP/TZ2P) summed up over the respective fragments for the hydrogen‐containing complexes and the free cluster in a.u.

B	q([B_12_H_12_])	q([B])	q(H)^a^	q(H)^b^
[]	−2.00		−0.10	
BH_3_	−1.62	−0.38	−0.02	
AlH_3_	−1.66	−0.34	−0.05	
GaH_3_	−1.71	−0.30	−0.07	
CF_2_	−1.73	−0.27	−0.05	
CCl_2_	−1.51	−0.49	−0.02	
CBr_2_	−1.46	−0.54	−0.02	
NH_3_	−1.94	−0.06	−0.10	
HF	−1.86	−0.14	−0.07	0.14
HCl	−1.84	−0.16	−0.07	0.08
HBr	−1.78	−0.22	−0.06	0.06

^a^ The hydrogen atom bonded to boron (X in Scheme 2).

^b^ The hydrogen atom bonded to halogen or nitrogen.

The trend in bond strengths is also found in the interaction energy (Δ*E*_int_) which describes the interaction of the deformed fragments or the bonding energy (Δ*E*_bond_) which includes this deformation energy (Δ*E*_prep_). The only difference is that the preparation energy is comparatively small for GaH_3_ and thus the resulting bond energy is much closer to BH_3_ than the interaction energy.

**FIGURE 1:**
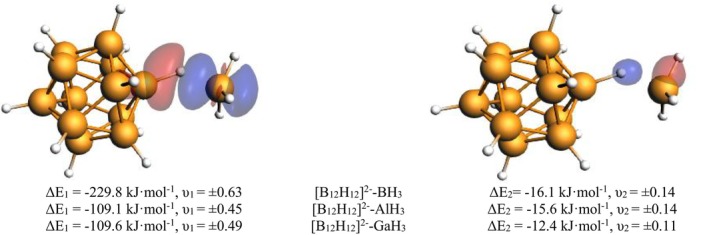
Most important deformation densities Δ*ρ*_1_ (left) and Δ*ρ*_2_ (right) of [B_12_H_12_]^2−^‐BH_3_ complexes from EDA‐NOCV analysis with energy values ΔE_i_ and eigenvalues υ_i_. Deformation density plots for Y = Al, Ga are found in the (Figure S1).

The character of this CIHB is then described by the EDA terms which show notable differences between the three Lewis acids. While bonding in the BH_3_ complex is dominated by the orbital interaction term (64% of the total attractive bonding contributions), the Al and Ga adducts show a balance between electrostatic and orbital contributions. The slightly larger electrostatic contribution is in line with a more typical EDA pattern seen in normal hydrogen bonding, where this term typically dominates [67, 68, 69]. The bonding in the BH_3_ complex shows a very similar electrostatic term in magnitude, but the share in the attractive interactions is smaller since the orbital term is much larger. This can be traced back to a much stronger donation of the B—H *σ*—bond towards the accepting boron atom at BH_3_ as shown by the EDA‐NOCV analysis (Figure 1a). The direction and nature of CT in CIHB is consistent with a previous study by Jabłoński [36]. This donation is a clear indication that the interpretation as CIHB is valid here since it quantitatively shows the interaction sketched in Scheme 2b. Also, the partial charges give some information (Table 3). While the charge of the hydrogen atom in the dianionic free [B_12_H_12_]^2−^ cluster is q(H) = −0.10, it gets less negative in the Lewis acid adducts, with the least negative hydrogen atom found for the BH_3_ adduct (q(H) = −0.02). This supports the notion of charge donation from the B—H bond towards the Lewis acid. For AlH_3_ and GaH_3_, this donation of the σ‐bond is smaller but still is the dominating orbital interaction in the system, as shown by the values for Δ*ρ*_1_ in Figure 1a. The energy gained through this interaction (Δ*E*_1_ in Table 2 and Figure 1a) nearly makes up all the orbital interaction term in all complexes. One more minor—but interesting—bonding contribution is found in all three complexes. As shown in Figure 1b, the second most important deformation density Δ*ρ*_2_ represents a hyperconjugation of the *σ*(Y—H) bond of the Lewis acid towards the σ*(B—H) bond of the boron cage. It is of similar magnitude in all complexes (Δ*E*_2_ in Table 2 and Figure 1b). The last term to be discussed is the Pauli repulsion (Δ*E*_Pauli_). It is difficult to compare this term directly for atoms from different rows of the periodic system since the orbital overlap is so vastly different [51]. Consequently, we see a large Pauli repulsion for the BH_3_ complex where we have large orbital overlap and a resulting large orbital term and short bond distance of *d*_H‐Y_ = 1.387 Å. For AlH_3_ now, the orbital overlap is much smaller (indicated by the much smaller orbital term) and the Pauli repulsion follows. Interestingly, for GaH_3_ we have—as expected—a further decrease in the orbital interaction but an increase in the Pauli repulsion. The latter trend is a major reason why GaH_3_ is weaker bonded than AlH_3_ and BH_3_.

Let us turn now to the trend with varying the hydrogen isotope. For all three complexes, the changes are rather minor. The bonding energy changes only up to a negligible amount of 0.1 kJ·mol^−1^. Thus, at first glance, one would assume that there are no geometric isotope effects. However, the EDA analysis reveals that indeed the character of the bond changes slightly. In all cases, the interaction energy gets slightly weaker while the preparation energy decreases by a similar amount, which results in the bonding energy staying unchanged when moving from H to D to T. This is a bit surprising since the bond length is shortened in all cases by up to 0.010 Å, which is small but significant. Thus, the bonds get shorter but not stronger. The EDA terms now reveal that the Pauli repulsion is increasing for X = D and X = T by 1.1–4.4 kJ·mol^−1^. This matches the shortening of the bond length. Note that we cannot make a statement about the differences in electronic structure between the hydrogen isotopes since, at the current stage, we limit our analysis to the geometric effect from the NEO‐DFT optimization. This increase in Pauli repulsion is then compensated for by different terms for different complexes. For the BH_3_ complex, we find an increase in electrostatic as well as orbital contributions. The latter effects stem mostly from a strengthening of the CIHB‐type interaction shown in Figure 1a. For the AlH_3_ complex, the electrostatic contribution even gets slightly less stabilizing, and all the bond strengthening comes from an increase in orbital contributions. Again, the first deformation density representing the donation of the *σ*(B—X) bond towards the Lewis acid gets a larger weight and provides more stabilization. For GaH_3_, the increase in orbital interaction is negligible for X = D and moderate for X = T (−1.1 kJ·mol^−1^). In this case, stabilization mainly stems from an increase in electrostatic interaction. Thus, although the bonding energy suggests that there are no changes in the CIHB to the boron cage when moving from H to D to T, the EDA reveals that we have small but distinct changes in the bonding character which result in zero change only as a sum. Furthermore, it is surprising that the influence of the isotope substitution is so different for these very similar complexes. We will now discuss whether these patterns are similar in the other complexes investigated.

**FIGURE 2:**
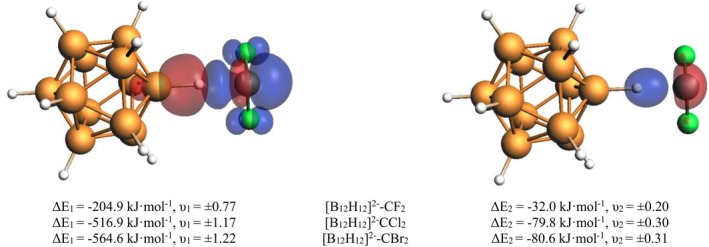
Most important deformation densities Δ*ρ*_1_ (left) and Δ*ρ*_2_ (right) of [B_12_H_12_]^2−^‐CF_2_ complexes from EDA‐NOCV analysis with energy values ΔE_i_ and eigenvalues υ_i_. Deformation density plots for Z = Cl, Br are found in the (Figure S2).

Next, we turn our attention to the carbene complexes [B_12_H_11_X]^2−^‐CZ_2_ (Z = F, Cl, Br) with the EDA data shown in Table 4. Again, we will discuss the trend with varying Z first before we turn to the isotope effects. The bond strength shows a clear trend of Br > Cl > F, which is seen in Δ*E*_bond_ as well as Δ*E*_int_. This is found despite the high preparation energies for the CCl_2_ and CBr_2_ adducts, which are in line with the short bond length. The bonding character is rather similar in all three adducts, with the attractive interactions being dominated by orbital interaction (62%–69%). This is another hint that the CIHB has a different bonding characteristic than the typical hydrogen bond, which is dominated by electrostatic interactions. The major contribution to the orbital interaction is, in all three complexes—like in the YH_3_ adducts discussed above—the *σ*(B—H) bond donation towards the accepting p‐orbital at the carbene carbon atom (Δ*Ε*_1_ in Figure 2). The second, but much less important, orbital interaction is the conjugation effect of the in‐plane sp^2^‐type lone pair at the carbon center towards the σ*(B—H) bond (Δ*Ε*_2_ in Figure 2). The Pauli repulsion term follows the trend of the other EDA terms. The EDA‐NOCV results indicate charge flow from negatively charged hydrogen of the boron clusters towards singlet carbenes, showing CIHB formation. These results are in good agreement with previous investigations on singlet carbenes interacting with negatively charged hydrogen [43, 70, 71]. The isotope effects are particularly pronounced here. We find a small effect on the bonding energy (< 1 kJ·mol^−1^) and a slightly larger effect on the interaction energy (up to 1.4 kJ·mol^−1^) which is following the trend in interaction strength of H > D > T in all three complexes. The major effect, however, is found for the EDA terms. While the Pauli repulsion is increasing in the series H > D > T, the same is true for the stabilizing orbital interaction term and—to a lesser extent—the electrostatic bonding contribution. However, as an overall effect, the increase in Pauli repulsion is dominating and thus makes the bond slightly weaker. The increase in the magnitude of all terms matches the decrease in bond length *d*_X‐C_. The stronger electron accepting character of the CZ_2_ fragments in comparison to the YH_3_ Lewis acids is also reflected in larger charge transfer, as indicated by an increase in the Hirshfeld charges (Table 3). Again, the donating H‐atom gets less negative in partial charge.

**TABLE 4:** EDA‐NOCV results (B3LYP/TZ2P) for [B_12_H_11_X]^2−^‐CZ_2_.

X=	Z = F			Z = Cl			Z = Br		
	H	D	T	H	D	T	H	D	T
		Δ*E* ^a^	Δ*E* ^a^		Δ*E* ^a^	Δ*E* ^a^		Δ*E* ^a^	Δ*E* ^a^
Δ*E*_int_	−55.5	0.7	1.1	−177.6	0.5	0.9	−210.2	0.7	1.4
Δ*E*_Pauli_	335.3	2.7	4.6	724.3	8.8	14.8	744.4	10.7	16.6
Δ*E*_elstat_ ^b^	−147.6 (38%)	−0.7	−1.2	−291.8 (32%)	−1.6	−3.1	−293.4 (31%)	−2.6	−3.9
Δ*E*_orb_ ^b^	−243.2 (62%)	−1.3	−2.2	−610.1 (68%)	−6.7	−11.0	−661.2 (69%)	−7.4	−11.0
Δ*E*_1_ ^c^	−204.9	−1.1	−1.9	−516.9	−6.1	−9.6	−564.6	−6.3	−9.7
Δ*E*_2_ ^c^	−32.0	−0.1	−0.3	−79.8	−0.6	−1.2	−80.6	−1.0	−1.5
Δ*E*_rest_ ^c^	−6.2	−0.0	−0.1	−13.4	−0.0	−0.1	−16.0	−0.1	−0.2
Δ*E*_prep_	22.3	−0.8	−1.3	81.0	−0.7	−1.2	92.7	−0.9	−1.7
Δ*E*_bond_	−33.1	−0.1	−0.2	−96.6	−0.2	−0.3	−117.5	−0.2	−0.3

		Δ*d* ^d^	Δ*d* ^d^		Δ*d* ^d^	Δ*d* ^d^		Δ*d* ^d^	Δ*d* ^d^
*d*_X‐C_	1.539	−0.004	−0.007	1.255	−0.006	−0.010	1.240	−0.006	−0.010

^a^ Δ*E* = E(D/T) – E(H). All energy terms in kJ·mol^−1^.

^b^ The percentage values give the contribution to the total attractive interaction Δ*E*_elstat_ + Δ*E*_orb_.

^c^ Contributions to Δ*E*_orb_ from EDA‐NOCV analysis (see Figure 2).

^d^ Δ*d* = *d*_D/T‐X_ – *d*_H‐X_. Distances in Å.

It is interesting to note that CF_2_ is very weakly attached compared to CCl_2_ and CBr_2_. This is in contrast to the Lewis acidity, where CF_2_ is the strongest Lewis acid due to the strong electron‐withdrawing effect of F combined with a small π‐back donation [72].

**FIGURE 3:**
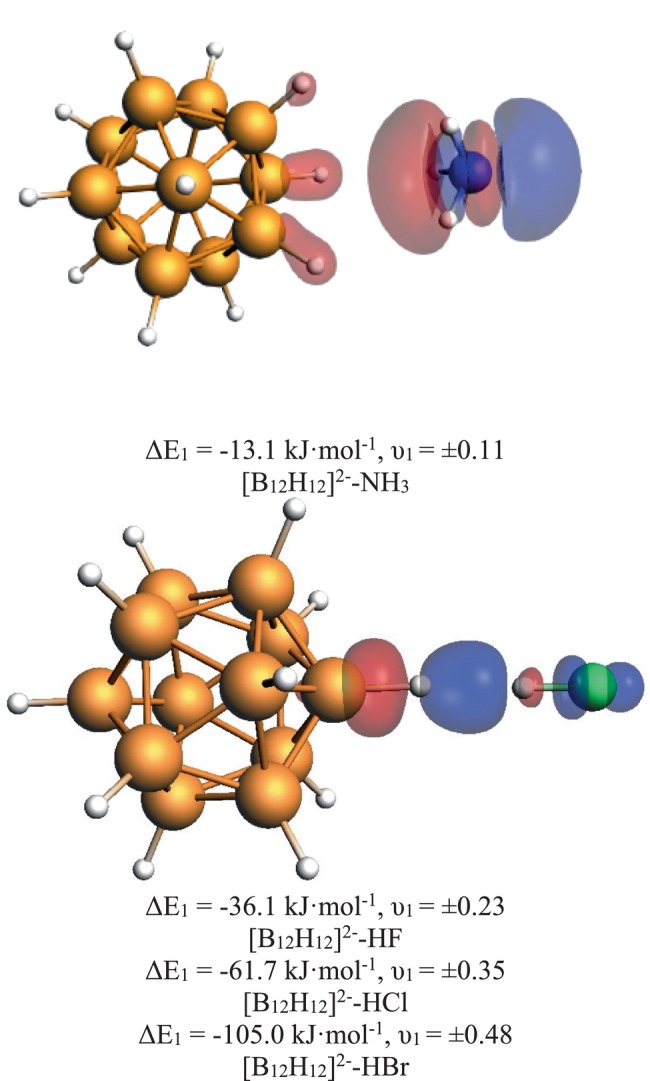
Most important deformation density Δ*ρ*_1_ of [B_12_H_12_]^2−^‐NH_3_ and [B_12_H_12_]^2−^‐HZ complexes from EDA‐NOCV analysis with energy values ΔE_1_ and eigenvalues υ_1_ for all complexes. Deformation density plots for Z = Cl and Br are shown in the (Figure S3).

As a last step, we now analyze the DHBs. We start with a discussion of the NH_3_ complex. The EDA results in Table 5 show that we have a rather small bond energy of Δ*E*_bond_ = −30 kJ·mol^−1^ in line with the long bond distance of 2.280 Å and the small fragment charge of −0.06 (Table 3). The only slight deformation of the fragments required for bonding—reflected in a small preparation energy—leads to a similar value for the interaction energy. Notably, this interaction is now dominated by electrostatic interaction (Δ*E*_elstat_ makes up 72% of the attractive interaction terms) as often found for normal hydrogen bonds. The isotope effects are completely different here compared to the CIHBs. While the EDA terms are virtually unaltered (the NOCV analysis also does not give much hint, Figure 3), the bond energy slightly decreases due to a reduction in preparation energy, in line with the shorter bond lengths found for the heavier isotopes.

**TABLE 5:** EDA‐NOCV results (B3LYP/TZ2P) for [B_12_H_11_X]^2−^‐NH_3_.

X=	H	D	T
		Δ*E* ^a^	Δ*E* ^a^
ΔE_int_	−33.4	0.0	0.1
ΔE_Pauli_	16.6	0.0	0.1
Δ*E*_elstat_ ^b^	−36.9 (72%)	0.0	0.0
Δ*E*_orb_ ^b^	−13.1 (28%)	0.0	0.0
Δ*E*_1_ ^c^	−13.1	0.0	0.0
Δ*E*_prep_	4.3	−0.2	−0.2
Δ*E*_bond_	−29.2	−0.1	−0.2

		Δ*d* ^d^	Δ*d* ^d^
*d*_X‐H_	2.280	−0.005	−0.009

^a^ Δ*E* = E(D/T) – *E*(H). All energy terms in kJ·mol^−1^.

^b^ The percentage values give the contribution to the total attractive interaction Δ*E*_elstat_ + Δ*E*_orb_.

^c^ Contributions to Δ*E*_orb_ from EDA‐NOCV analysis (see Figure 3).

^d^ Δ*d* = *d*_D/T‐X_ – *d*_H‐X_. Distances in Å.

It is now instructive to compare this to the much stronger DHBs in the complexes [B_12_H_11_X]^2−^‐HZ (where Z = F, Cl, Br and X = H, D or T) shown in Table 6. The trends are more complex compared to the CIHBs. In bonding energy, the HF complex is the strongest and HCl and HBr show similar bond energy. Regarding intrinsic bond energy (Δ*E*_int_), however, the HBr complex is most strongly bonded. This is a result of the strong increase in orbital interaction energy. While the Δ*E*_orb_ term is contributing only 41.3% in the HF complex, it is nearly 65.2% in the HBr complex. This is a clear indication that the intrinsic DHB strength in the latter complex is the strongest as also shown in the NOCV analysis with deformation density Δ*ρ*_1_ in Figure 3. The trend in partial charges are less pronounced for this set of compounds (Table 3). It is surprising that the bond distance between the differently polarized H atoms in the DHB are getting so much shorter in the series HF > HCl > HBr. The sharp increase in Pauli repulsion and also the much higher preparation energy, however, lead to rather similar bond energies which do not match this bond distance trend.

**TABLE 6:** EDA‐NOCV results (B3LYP/TZ2P) for [B_12_H_11_X]^2−^‐HZ.

X=	Z = F			Z = Cl			Z = Br		
	H	D	T	H	D	T	H	D	T
		Δ*E* ^a^	Δ*E* ^a^		Δ*E* ^a^	Δ*E* ^a^		Δ*E* ^a^	Δ*E* ^a^
Δ*E*_int_	−69.8	0.0	0.0	−65.4	0.1	0.4	−78.0	−0.1	0.1
Δ*E*_Pauli_	38.5	0.8	1.2	69.7	0.1	−0.0	115.5	2.5	3.9
Δ*E*_elstat_ ^b^	−64.6 (59.7%)	−0.2	−0.3	−60.7 (44.9%)	0.0	0.1	−67.1 (34.8%)	−0.5	−0.7
Δ*E*_orb_ ^b^	−43.7 (41.3%)	−0.5	−0.9	−74.4 (55.1%)	0.0	0.3	−126.0 (65.2%)	−2.3	−3.1
Δ*E*_1_ ^c^	−36.1	−0.4	−0.7	−61.7	0.1	0.3	−105.0	−1.6	−2.4
Δ*E*_2_ ^c^		0.0	0.0		0.0	0.0	−15.7	−0.3	−0.5
Δ*E*_rest_ ^c^	−7.7	−0.1	−0.2	−12.7	0.0	0.0	−5.4	−0.4	−0.1
Δ*E*_prep_	4.0	−0.1	−0.2	9.1	−0.2	−0.5	19.0	−0.1	−0.3
Δ*E*_bond_	−65.8	−0.1	−0.2	−56.3	−0.1	−0.1	−58.9	−0.2	−0.2

		Δ*d* ^d^	Δ*d* ^d^		Δ*d* ^d^	Δ*d* ^d^		Δ*d* ^d^	Δ*d* ^d^
*d*_X‐H_	1.432	−0.007	−0.011	1.362	−0.001	−0.001	1.218	−0.007	−0.013

^a^ Δ*E* = E(D/T) – E(H). All energy terms in kJ·mol^−1^.

^b^ The percentage values give the contribution to the total attractive interaction Δ*E*_elstat_ + Δ*E*_orb_.

^c^ Contributions to Δ*E*_orb_ from EDA‐NOCV analysis (see Figure 3).

^d^ Δ*d* = *d*_D/T‐X_ – *d*_H‐X_. Distances in Å.

The isotope effects are quite small here. Although most terms do only change by a few tenths of kJ·mol^−1^, the Pauli repulsion and orbital interaction energy get a bit larger each for the HBr complexes. Similar trends are found for HF, while for the HCl complex the EDA terms stay quite constant. This matches the very small changes in bond lengths for the latter complex. The positive partial charge at the hydrogen atom of the HZ fragment underlines the DHB character of the bond (Table 3).

## 4 Conclusion

We used NEO‐DFT to capture primary and secondary geometric isotope effects on a set of compounds representing charge‐inverted hydrogen bonds (CIHB) as well as dihydrogen bonds (DHB). The dianionic but electrophilic boron cage [B_12_H_12_]^2−^ is used as a bonding partner exhibiting a negatively polarized hydrogen atom in the B—H bond. A set of Lewis acids (AlH_3_, BH_3_ and GaH_3_) as well as carbenes (CF_2_, CCl_2_ and CBr_2_) are used as bonding partners for the CIHBs. The isotope effects on the structure are shown to be quite systematic, with bond lengths decreasing in the series H > D > T for the primary as well as the secondary effect. DHBs are analyzed with the bonding partners NH_3_, HF, HCl, and HBr.

The EDA analysis combined with Hirshfeld partial charge analysis clearly supports the bonding interpretation in all cases. However, it also shows that the bonding character can vary significantly within the same family of compounds. The EDA also gives hints on the influence of the geometric isotope effects on the electronic structure. Although the changes in energy terms are rather small throughout, they show distinct trends. And surprisingly, the changes can again be different within the same compound class. While in some cases an increase in electrostatic attraction is the major effect (HB, DHB), for CIHBs the orbital interaction increases significantly. Also, the change in Pauli repulsion can determine the trend.

This study gives a first idea on how a systematic investigation of geometric isotope effects on electronic structure can be analyzed with quantitative bonding analysis methods. In the next step, we want to extend this study to a broader set of interactions. By targeting more strongly bound systems, we expect that the isotope effects will increase and the resulting changes in the electronic structure will be more pronounced. In the long term, a direct analysis of the electronic effects of isotope substitution would be desirable. However, this would require a significant extension of the currently available methods.

## Conflicts of Interest

The authors declare no conflicts of interest.

## Supporting information


**Figure S1:** Most important deformation densities Δ*ρ* of [B H]^2−^‐YH complexes from EDA‐NOCV analysis with energy values Δ*E* and eigenvalues *υ*.
**Figure S2:** Most important deformation densities Δ*ρ* of [B H]^2−^‐CZ complexes from EDA‐NOCV analysis with energy values Δ*E* and eigenvalues *υ*.
**Figure S3:** The shape of deformation densities of [B H]^2−^‐NH and [B H]^2—^HZ complexes.

## Data Availability

The data that support the findings of this study are openly available in Zenodo at https://zenodo.org/records/15642293.
