# Supplementary material for: Geometrical Isotope Effects on Chemical Bonding in Hydrogen Bonded Systems: Combining Nuclear‐Electronic Orbital DFT and Energy Decomposition Analysis
Source: J Comput Chem. 2025 Sep 11;46(24):e70226. doi: 10.1002/jcc.70226 (PMC12423738; doi:10.1002/jcc.70226)
Supplement: Supplementary file 1 — Figure S1: Most important deformation densities Δρ of [B H]2−‐YH complexes from EDA‐NOCV analysis with energy values ΔE and eigenvalues υ. Figure S2: Most important deformation densities Δρ of [B H]2−‐CZ complexes from EDA‐NOCV analysis with energy values ΔE and eigenvalues υ. Figure S3: The shape of deformation densities of [B H]2−‐NH and [B H]2—HZ complexes. [file JCC-46-0-s001.pdf]

Geometrical isotope effects on chemical bonding in hydrogen bonded systems: Combining nuclear-electronic orbital DFT and energy decomposition analysis.

Raza Ullah Khan, Ralf Tonner-Zech*

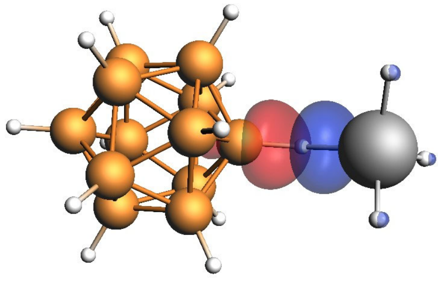

$[B_{12}H_{12}]^{2-}$-AlH_3

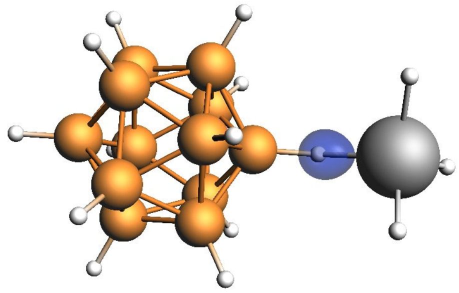

$\Delta\rho_1$:ΔE_1 = -109.1 kJ/mol, υ_1= ±0.45

$\Delta\rho_2$:ΔE_2 = -15.6 kJ/mol, υ_2= ±0.14

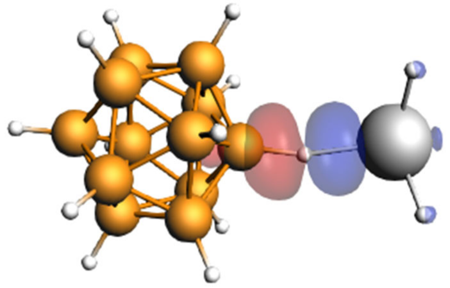

$[B_{12}H_{12}]^{2-}$-GaH_3

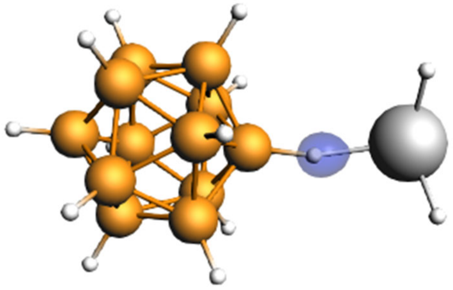

$\Delta\rho_1$:ΔE_1 = -109.6 kJ/mol, υ_1= ±0.49

$\Delta\rho_2$:ΔE_2 = -12.4 kJ/mol, υ_2= ±0.11

Figure S1. Most important deformation densities $\Delta\rho$ of $[B_{12}H_{12}]^{2-}$-YH_3 complexes from EDA-NOCV analysis with energy values ΔE and eigenvalues υ

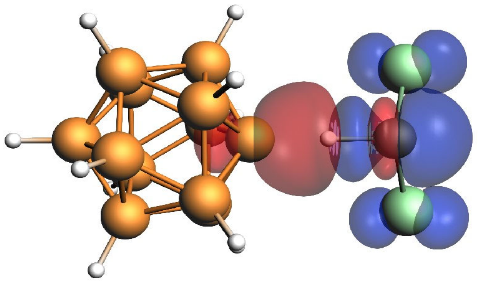

$[B_{12}H_{12}]^{2-}CCl_2$

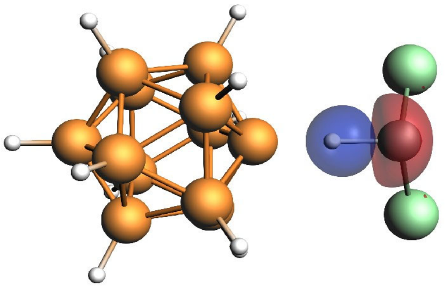

$\Delta\rho_1$:ΔE_1= -516.9 kJ/mol, υ_1=±1.17

$\Delta\rho_2$:ΔE_2 = 79.8 kJ/mol, υ_2=±0.30

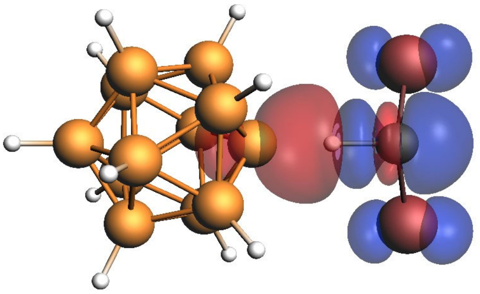

$[B_{12}H_{12}]^{2-}$-CBr_2

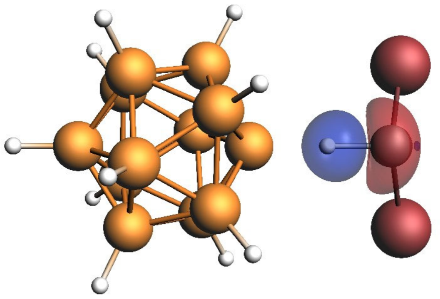

$\Delta\rho_1$:ΔE_1= -564.6 kJ/mol, υ_1=±1.22

$\Delta\rho_2$:ΔE_2= -80.6 kJ/mol, υ_2=±0.31

Figure S2. Most important deformation densities Δρ of $[B_{12}H_{12}]^{2-}$-CZ_2 complexes from EDA-NOCV analysis with energy values ΔE and eigenvalues υ.

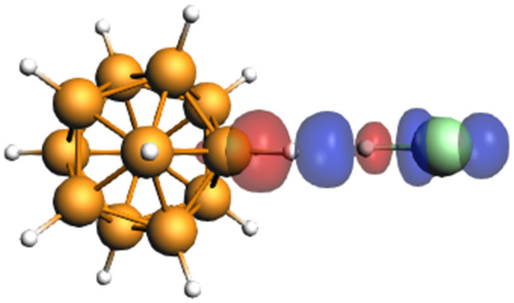

Δρ1:ΔE1 = -61.7 kJ/mol, υ1=±0.35 $[B_{12}H_{12}]^{2-}$-HCl

$[B_{12}H_{12}]^{2-}$-HCl

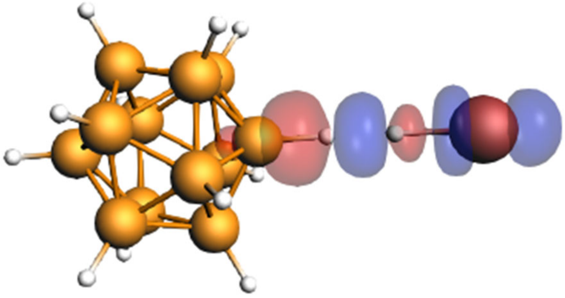

Δρ1:ΔE1 = -105.3 kJ/mol, υ1=±0.48

$[B_{12}H_{12}]^{2-}$-HBr

Figure S3. The shape of deformation densities of $[B_{12}H_{12}]^{2-}$-NH_3 and $[B_{12}H_{12}]^{2-}$-HZ complexes.
